# Immune Responses Following Locoregional Treatment for Hepatocellular Carcinoma: Possible Roles of Adjuvant Immunotherapy

**DOI:** 10.3390/pharmaceutics13091387

**Published:** 2021-09-02

**Authors:** Ji-Won Han, Seung-Kew Yoon

**Affiliations:** 1 Division of Gastroenterology and Hepatology, Department of Internal Medicine, College of Medicine, Seoul St. Mary’s Hospital, The Catholic University of Korea, Seoul 06591, Korea; tmznjf@catholic.ac.kr; 2 The Catholic University Liver Research Center, College of Medicine, The Catholic University of Korea, Seoul 06591, Korea

**Keywords:** HCC, immunotherapy, locoregional treatment, adjuvant treatment

## Abstract

Hepatocellular carcinoma (HCC) is a common cause of cancer-related deaths worldwide. Unlike other types of cancer, HCC can be treated with locoregional treatments (LRTs) such as radiofrequency ablation (RFA) or transarterial chemoembolization (TACE). However, recurrences following LRTs are common, and strategies to improve long-term outcomes need to be developed. The exhaustion of anti-tumor immunity in HCC has been well established in many reports and the immunomodulatory effects of LRTs (enhancement of tumor antigen-specific T cell responses after RFA, reduction of effector regulatory T cells after TACE) have also been reported in several previous studies. However, a comprehensive review of previous studies and the possible roles of immunotherapy following LRTs in HCC are not known. In this review, we discuss the immunological evidence of current clinical trials using LRTs and combined immunotherapies, and the possible role of this strategy.

## 1. Introduction

Hepatocellular carcinoma (HCC) is the fourth most common cause of cancer-related deaths worldwide, and its incidence is increasing despite recent advances in treatment and surveillance strategies for chronic liver diseases, including viral hepatitis [1]. Although HCC distribution varies per region, the basic principles for HCC treatment are based on the Barcelona Clinic Liver Cancer (BCLC) algorithm [2]. Liver resection or liver transplantation is indicated in early stage diseases such as single or three nodules less than 3 cm. However, early stage HCCs can also be treated with local ablation methods such as radiofrequency ablation (RFA), microwave ablation (MWA), percutaneous ethanol injection (PEI), or cryoablation, especially in patients not eligible for surgery. Intermediate-stage HCCs have been treated with transarterial chemoembolization (TACE), which uses tumor-feeding arteries to infuse cytotoxic chemotherapeutic agents, followed by immediate embolization. These various options for locoregional treatments (LRTs) might be unique for HCCs compared to other cancer types. The options are mainly based on image-guided liver tumor-directed procedures, and most HCC patients undergo LRTs at least once during the illness [3].

LRTs have a substantial recurrence rate; the median overall survival (OS) was 60 months and a 50–70% recurrence rate after successful RFA within 20–30 months has been reported [4]. Furthermore, TACE has an objective response rate (ORR) of 52.5% [5] and median survival of 26–40 months [6,7]. Thus, complementary strategies following LRT are needed to improve the clinical outcomes.

Since its approval in 2007, sorafenib has been the only option for the systemic treatment of advanced HCC. Other systemic treatments have recently been developed for advanced stage, including novel tyrosine kinase inhibitors (TKIs) such as lenvatinib as a first-line treatment and regorafenib or cabozantinib as second-line treatment. Therefore, a combination of TKIs and LRTs has been investigated to improve clinical outcomes. However, sorafenib followed by resection or ablation (STORM trial [8]) has failed to improve recurrence-free survival in the adjuvant setting compared with a placebo. Similarly, TKIs following TACE failed to improve clinical outcomes in several previous trials [9,10,11]. Based on this evidence, the current consensus does not recommend adjuvant chemotherapy following LRTs.

Immune checkpoint blockade (ICB), such as nivolumab is indicated for unresectable HCC. Nevertheless, monotherapies using ICBs failed to show a marked effect in HCC compared to other tumors, as shown in the recent clinical trials [12]. The liver is an immune-tolerant organ, and multiple cellular components such as endothelial cells, Kupffer cells, and Treg cells limit effector T-cell responses [13]. Additionally, liver-resident T cells, which contribute to the rapid local immune responses, are functionally suppressed, compared to T cells within other organs [14]. An increase of immunosuppressive immune-cell populations, including Treg cells, M2 macrophages, or MDSCs in patients with HCC might synergize to the failure of ICB-monotherapy [15]. Tumor intrinsic mechanisms of ICB-resistance, such as upregulation of PD-L1, downregulation of MHC-I, or increase of oncogenic, β-catenin signaling pathway might also contribute to the failure of ICB-monotherapy [15]. Therefore, various strategies to overcome mechanisms of insufficient response to the ICBs in HCC patients have been examined. A combination regimen of atezolizumab plus bevacizumab recently showed better clinical outcomes as first-line treatment than sorafenib [16]. Furthermore, another combination regimen, pembrolizumab plus lenvatinib, is currently under clinical trial and has promising clinical outcomes in the early phase of clinical trials [17]. Whether this immunotherapeutic strategy plays a role in the adjuvant setting following LRTs in patients with HCC remains unclear. The immunological characteristics of HCC and the immunological changes following LRTs have recently been investigated. In a previous report, adjuvant therapy using cytokine-induced killer cells prolonged recurrence-free survival after curative therapy including resection, RFA, or PEI [18], indicating that the augmentation of immune responses following LRTs might benefit the clinical outcome of HCC.

A previous report suggested that the recent advancement in the surveillance and treatment modalities during the 15 years has led to early detection of HCC, improved treatment efficacies, and prolonged patients’ survival [19]. These findings suggest that the advances in the clinical outcome of HCC could be achieved from more efforts to improve treatment modalities including combination treatments.

In this manuscript, we briefly review the immunological characteristics of HCC, including rationales for ICBs, and summarize the changes after each LRT. Based on these summaries, current trials on adjuvant immunotherapies following LRTs in HCC are described.

## 2. Evidence of Current ICBs for HCC

**Figure 1:**
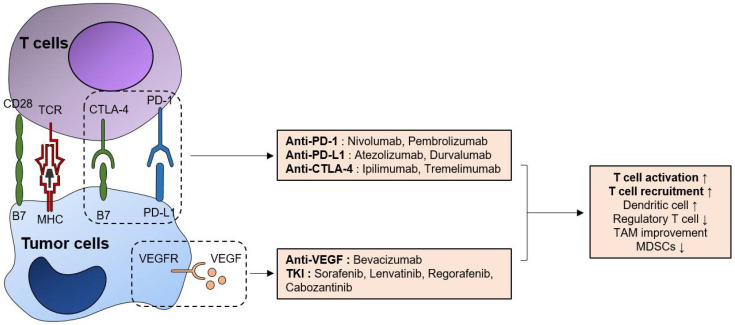
Immunologic effects of currently available systemic agents for HCC treatment. Currently available ICBs, such as anti-PD-1, anti-PD-L1, and anti-CTLA-4 can activate anti-tumor T-cell responses via the augmentation of TCR-mediated T-cell activation (signal 1) and CD28-B7 interaction (signal 2). Additionally, anti-VEGF bevacizumab also has T-cell functional enhancing effects, both directly and indirectly by regulating immunosuppressive cell populations such as tumor-associated macrophages (TAMs) and MDSCs. TKIs have various impacts on the anti-tumor immune responses, but they all have anti-VEGF effects and augment anti-tumor immune responses by inhibiting VEGF-VEGFR pathway.

Figure 1 depicts current available drugs that augment anti-tumor immune responses, including ICBs, anti-VEGF, and TKIs. Additionally, we briefly summarized their mechanism of action and their targets. The current strategy for immunotherapy is based on blocking immune checkpoint molecules such as programmed cell death protein-1 (PD-1) and cytotoxic T-lymphocyte-associated protein-4 (CTLA-4) expressed on T cells, which are induced by chronic tumor-antigen stimulation. The dysfunctional tumor-immune microenvironment (TIME), including T cell exhaustion or dysfunction and other associated factors, has been actively investigated in various types of cancers as well as in HCC.

Currently available ICBs are anti-PD-1 (nivolumab), anti-PD-1 plus anti-CTLA-4 (pembrolizumab plus ipilimumab), and anti-PD-L1 (atezolizumab) combined with anti-vascular endothelial growth factor (VEGF) bevacizumab [20]. CD8^+^ T cells, which are the main effectors of anti-tumor adaptive immune responses, upregulate immune checkpoint molecules such as PD-1, in addition to CTLA-4, hepatitis A virus cellular receptor 2 (TIM-3), and lymphocyte activating gene 3 (LAG-3) in patients with HCC, and blocking them in vitro significantly restored the function of exhausted CD8^+^ T cells [21,22]. Furthermore, PD-1 upregulation in intratumoral T cells has been described in several previous studies and might be associated with clinical outcomes [23]. For example, patients with low intratumoral PD-1^high^ signatures had better median OS in the TCGA cohort [21]. Furthermore, PD-1-expressing B cells suppress T cell responses via IL-10 [24]. Therefore, anti-PD-1 agents mainly target CD8^+^ T cell responses, thereby augmenting the adaptive immune responses against tumors. Based on this evidence, compared with sorafenib, nivolumab in patients with advanced HCC as a first-line treatment tended to improve survival [25] in a phase I/II clinical trial, although it did not reach the primary endpoint (NCT02576509).

In addition, PD-L1-expressing HLA-DR^+^ macrophages were associated with poor survival [26] and PD-L1-expressing IgA^+^IL-10^+^ B cells suppress anti-tumor CD8^+^ T cell responses [27]. Furthermore, circulating tumor cells express PD-L1 in HCC and are significantly associated with OS in patients receiving anti-PD-1 treatment [28]. T cells residing in the liver highly express PD-1 [13,29], and liver-resident NK cells restrict T cell responses in a PD-1-PD-L1-dependent manner [30]. Therefore, blocking PD-L1 and PD-1 may be an important immunotherapeutic strategy. Additionally, in a phase III trial of anti-PD-L1, atezolizumab plus bevacizumab was significantly superior to sorafenib as a first-line treatment in advanced unresectable HCC in terms of 12-month OS (67.2% versus 54.6%) and progression-free survival (6.8 months versus 4.3 months) [16], which might be a significant breakthrough in chemotherapy for HCC.

In addition to chronic antigenic stimulation and exhaustion of CD8^+^ T cells, other immune cells contribute to weak anti-tumor CD8^+^ T cell responses in HCC. CD4^+^CD25^+^FoxP3^+^ regulatory T (Treg) cells exhibit on their surface both CTLA-4 and PD-1 and can be a target cell population of ICBs [31]. They are increased in the HCC microenvironment and impair CD8^+^ T cell-responses [32,33]. Intratumoral activated Treg cells constrain anti-tumor immunity in patients with HCC [34]. Among various mechanisms of immune-suppressive function of Treg cells, IL-10 and TGF-β secreted by them have been proven to induce functional impairment of T-cell responses [35]. Therefore, this population represents a direct target of new immunotherapies. CTLA-4-expressing CD14^+^ dendritic cells (DCs) are also associated with the attenuation of anti-tumor T cell responses due to indoleamine 2, 3-dioxygenase (IDO) and IL-10 production [36]. As exhausted effector T cells also upregulate CTLA-4, it might be an immunotherapeutic target for HCC. Based on this evidence, an anti-CTLA-4 blockade using ipilimumab plus nivolumab was also tested in sorafenib-treated HCC patients (CheckMate 040) as a phase I/II trial and showed a promising ORR of 31% with manageable adverse events (NCT01658878).

The results of previous basic and clinical studies indicate that a better understanding of the immunosuppressive environment in HCC may provide novel therapeutic strategies for advanced stage HCC. However, adjuvant treatment following LRT or surgical treatment has not been established in early to intermediate stage HCC. The promising results of ICBs in patients with advanced HCC indicate possible options for adjuvant treatment in early to intermediate stage HCC. In melanoma, ICBs have been studied and approved as adjuvant treatments. The use of adjuvant pembrolizumab in stage III resected patients [37] and nivolumab in stage III and IV resected patients [38] reduced the risk of relapse by 40–50% and was approved by the FDA. ICBs could also be considered as adjuvant treatments, especially in patients receiving LRTs, to improve clinical outcomes. Therefore, the immunological changes following each LRT and their possible mechanisms were reviewed to summarize the experimental evidence for the current trials of adjuvant immunotherapies in HCC patients with LRTs.

Recently, the synergistic effect of systemic therapy and ICBs has been actively reported. VEGF/VEGFR signaling is associated with the immunosuppressive tumor microenvironment, in addition to its primary role in angiogenesis [39]. For example, VEGF/VEGFR signaling can suppress the function and differentiation of DCs, induce MDSCs [40]. Furthermore, VEGF can directly induce TOX-dependent T-cell exhaustion [41]. Based on these mechanisms, a murine study showed that dual anti-PD-1/VEGFR therapy suppressed tumor growth and survival, via vascular normalization and enhancing anti-tumor immune responses [42].

Currently available TKIs also have immunomodulatory effects. Firstly, used TKI sorafenib has been studied for its various impacts on immune cells. It can improve anti-tumor immune responses by regulating macrophages [43] and MDSCs [44], enhancing effector T-cell responses [45], and reducing Treg frequency [46]. Especially, regorafenib demonstrated anti-immunosuppressive properties, as well as promoting anti-tumor immunity [47]. In addition to improving DC and T cell responses, and modulating TAMs and Treg cells by VEGF inhibition, regorafenib has additional advantages in targeting immunosuppressive TIE2-expressing monocyte/macrophages and endothelial cells [47]. Similarly, other TKIs such as lenvatinib and cabozantib can augment anti-tumor immune responses [48]. Therefore, combination strategies of ICBs and anti-VEGF or TKIs have actively under the clinical trial in patients with unresectable HCC, as well as in patients who underwent LRT as an adjuvant treatment.

## 3. Effects of Cell Death in Anti-Tumor Immune Responses Induced by LRTs

Several mechanisms have been suggested regarding the effects of LRTs on anti-tumor immune responses; cancer cell death caused by LRTs is closely associated with immunomodulatory effects. Both apoptosis and necrosis of tumor cells can be induced by LRTs. In general, necrosis is known to be immunogenic, because the plasma membrane is broken, and necrotic cells release cellular contents including cancer-specific antigens and DAMPs [49]. This phenomenon augments innate and adaptive immune responses via necroinflammation [50]. Conversely, apoptosis has been known to be non-immunogenic, because that is a process of programmed cell death, in which the plasma membrane is not disrupted [49]. Nevertheless, previous reports have also implicated that certain types of apoptosis could be immunogenic [49]. For example, melphalan-induced cancer cell apoptosis was associated with the secretion of proinflammatory cytokines [51]. Additionally, the exposure of cardiac glycosides induced HMGB-1, a representative DAMP, which resulted in immunogenic cancer cell apoptosis [52]. Thus, in addition to the type of cell death, whether LRT-related cell death is immunogenic or non-immunogenic has been investigated.

The release of tumor antigens due to cell death and subsequent changes in antigen-presenting cells (APCs) and effector immune cells are the main processes responsible for the changes in anti-tumor immune responses after LRTs [53]. Tumor cell death caused by RFA releases tumor antigens for DCs, which induce anti-tumor immune responses [54]. In another study, RFA induced APC infiltration and effective anti-tumor immune responses [55].

The type of cell death might also be important in inducing an anti-tumor immune response. In a previous study, the apoptosis of tumor cells due to irradiation (not injection) of necrotic tumor cells augmented the effects of tumor cell vaccines [56]. In another report, necrosis of tumor cells failed to induce CD8^+^ T cell responses and was associated with the release of peptidases [57]. LRTs induce tumor cell necrosis by thermal injury, but may also induce tumor cell apoptosis. For example, subtotal thermal injury in the transitional zone of RFA caused apoptosis and increased expression of heat shock protein 70, which might be associated with enhanced immunogenicity [58]. In another study, tumor cell apoptosis was shown to be activated by RFA in the early period [59]. Most of the tumor cells in HCC patients who underwent TACE before surgical resection were necrotic, and 11% of the tumor cells exhibited apoptosis [60]. These findings indicated that immunogenic cell death and apoptosis could also be induced by LRTs, as well as necrosis, which is considered as non-immunogenic cell death. However, considering the high recurrence rate even after successful LRTs, a strategy to augment the anti-tumor immune responses following LRTs might also be necessary to improve tumor surveillance.

Doxorubicin, which is the most commonly used agent in TACE, can induce immunogenic cell death. The effects on cell death can differ with the types of chemotherapeutics; doxorubicin can promote immunologic cell death, but cisplatin cannot [61]. Conversely, doxorubicin in a transplantable murine lymphoma model expanded myeloid-derived suppressor cells (MDSCs) and resulted in PD-1/PD-L1-dependent immunosuppression [62], indicating that targeting immune checkpoint molecules following TACE with doxorubicin is helpful for effective anti-tumor responses.

## 4. Immunological Changes Following LRTs in HCC

**Figure 2:**
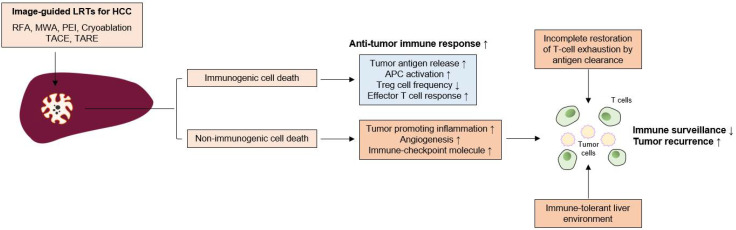
Insufficient immune surveillance after LRTs for HCC. Image-guided locoregional treatments (LRTs) such as radiofrequency ablation (RFA), microwave ablation (MWA), percutaneous ethanol injection (PEI), cryoablation, transarterial chemoembolization (TACE), and transarterial radioembolization (TARE) cause tumor-cell necrosis or apoptosis. LRT can induce immunogenic cell death, which can be characterized by increased tumor-antigen release, antigen presenting cell (APC) activation, and decrease of regulatory T (Treg) cell frequency. These phenomena result in the augmentation of anti-tumor T cell responses and tumor surveillance. However, LRT can also induce a non-immunogenic cell death, which increases the tumor-promoting inflammation, angiogenesis, and the expression of immune checkpoint molecules. Furthermore, incomplete T cell restoration despite antigen clearance and immune-tolerant liver environment might also affect the attenuation of immune surveillance; these factors might be associated with frequent tumor recurrence even after successful LRTs for HCC.

Evidence regarding immunological changes based on the HCC stage is lacking; however, in the analysis of the exhausted, dysfunctional immune cells within the tumor and adjacent liver, the surgical samples used in most immunological studies were from relatively early stage HCC patients. Immune dysfunction may begin in the early stage of tumor development. In a murine model, tumor-specific T cell exhaustion was initiated at the early pre-malignant phase of tumorigenesis [63]. In a previous study with human samples, PD-1^+^-exhausted CD8^+^ T cells within the tumor were also increased in patients with stage I and stage II or III HCC [64]. Furthermore, peritumoral CD8^+^ T cells exhibit exhausted features [64]. Although further investigation is necessary, these functional and phenotypic features of T cell exhaustion caused by chronic antigenic stimulation might be restored, but do not appear fully recovered by complete antigen clearance [65]. Thus, immune dysfunction can remain after successful LRT for HCC and LRT alone may induce limited antitumor immune responses. In the following sections, the previous studies in which the immunological changes caused by LRT in HCC were investigated are introduced and summarized in Figure 2.

### 4.1. Local Ablative Therapies

RFA is the most studied LRT in terms of immunological changes following LRTs. The immunomodulatory effect of RFA may be associated with tumor cell death caused by coagulative necrosis and apoptosis [66]. Cryoablation also showed inflammatory and coagulative responses, which may be associated with immune activation [67].

The increased number of tumor-specific T cells is associated with reduced tumor recurrence following RFA [68]. Similarly, MWA also increased the peripheral T cell number and increased the Th1/Th2 cytokine ratio [69]. A rat model of HCC showed that RFA treatment of the tumor in the left hepatic lobe enhanced T cell infiltration in the right lobe, thereby reducing tumor growth [70]. RFA enhanced T cell trafficking to the liver, which possesses an effector function against tumor antigens, but has no effect on B cell responses [71]. Notably, T cell activation caused by RFA tended to be stronger than that caused by surgical resection [72]. The activation of T cells may also be associated with DC activation. In HCC patients treated with RFA or PEI, activation of myeloid DCs and increased serum levels of tumor necrosis factor-α (TNF-α) and IL-1β were observed [73]. RFA or cryoablation also increases antigen-loaded DCs within the lymph nodes [54]. RFA also increased Th1 cytokines (interferon-γ and TNF-α) in HCC patients, which might be associated with enhanced T cell responses [74]. These results indicated that RFA can augment T cell infiltration into the liver and anti-tumor T cell responses in number and strength.

A 50–70% recurrence rate after successful RFA within 20–30 months has been reported in several studies [4]. In a previous study, tumor-specific T cell responses were enhanced by RFA in a liver cancer rabbit model, including circulating T cell activation and intrahepatic T cell infiltration; however, a high recurrence rate was also observed [75]. This finding was further validated in human subjects, which showed that immune activation following RFA was not associated with the prevention of HCC recurrence [76]. In addition, circulating PD-L1/PD-1 expression was significantly increased after cryoablation, correlating with poor clinical outcomes [77]. Increased Th17 cells after MWA have been shown to be a risk factor for tumor recurrence [78]. These findings indicate that the augmentation of immune responses following RFA is insufficient to prevent tumor recurrence.

The Toll-like receptor 9 agonist CpG increased anti-tumor T cell responses following RFA and prolonged survival and reduced new tumor growth [79]. A weak tumor-specific T cell response induced by RFA was potentiated by an anti-CTLA-4 blockade [80]. In another study, anti-CTLA-4 blockade or Treg cell depletion after RFA or cryoablation increased tumor-specific T cell number and cytokine secretion [54]. Interestingly, RFA of liver metastases increased T cell infiltration but also increased PD-L1 expression in primary tumors, which was overcome by anti-PD-1 blockade [81]. Furthermore, a mouse model showed that the administration of CC chemokine ligand 3 after RFA potentiated tumor-specific T cell responses via CD11c^+^cells in a CCR1-dependent manner [82]. In addition to the activation of T cell responses, RFA reduces the frequency of MDSCs, which is associated with a better recurrence-free survival [83]. These findings indicate that further immunotherapeutic strategies after local ablation treatments is helpful for improving clinical outcomes and enhancing tumor surveillance by effective immune responses, and strategies beyond targeting immune checkpoint molecules need to be investigated.

### 4.2. Transarterial Therapies

#### 4.2.1. TACE

TACE is the preferred method for treating intermediate-stage, unresectable HCCs. Despite procedural advancement, the clinical outcome has not been satisfactory and the median OS, which is affected by tumor size, tumor markers, liver function, and vascular invasion, was 19.9 months based on a recent report [84]. Similar to local ablative treatments, immune dysfunction may remain even after successful treatment. Furthermore, in a previous study with human samples, PD-1^+^-exhausted CD8^+^ T cells in the tumor were more increased in stage II or III patients than in stage I patients [64], indicating that T-cell exhaustion is more advanced in patients receiving TACE than in patients receiving local ablation treatments. As TACE is not a curative treatment and a small number of tumor cells can remain in circulation and within the liver, surveillance and clearance by immune cells and their augmentation using ICBs could have clinical benefit [13] and clinical trials are ongoing.

The changes in the composition or function of immune cells after TACE remain to be elucidated, and whether the peripheral T cell population might be affected by TACE has been investigated in only a few studies. Growing evidence indicates that tumor necrosis caused by TACE induces immunological activation. For example, the inflammatory cytokine IL-6 significantly increased early after TACE [85]. However, Th2 cytokines (IL-4, IL-5, or suppressive cytokine IL-10) were also increased in the late phase after TACE, indicating that the immunological changes caused by TACE are complicated processes.

Immunologic cell death markers, such as high mobility group box 1 (HMGB1) and soluble receptor for advanced glycation end products (sRAGE), inducers of PD-L1 in tumor cells, are increased by TACE in HCC patients [86,87]. In a previous report, the peripheral CD4^+^/CD8^+^ T cell ratio was significantly lower in patients with HCC prior to TACE than in healthy volunteers and was increased 1 month after TACE [88,89], although its effect on anti-tumor immunity was not investigated in those studies. However, in another study, TACE increased the frequency of tumor-specific CD4^+^ T cells, which was associated with improved clinical outcomes [90]. Notably, a recent report showed that baseline PD-L1 expression within peripheral blood mononuclear cells was significantly higher in poor TACE responders, which were defined as SD or PD by RECIST criteria, and patients with high PD-L1 expression of PBMCs after TACE showed poor OS [91]. Additionally, TACE increases PD-1 expression in peripheral mononuclear cells [91]. These findings indicate that targeting the PD-1-PD-L1 axis might benefit the clinical outcome after TACE in terms of response rate and survival, although the effects of PD-1 expression on anti-tumor immunity before and after TACE remain unclear.

Several studies have indicated that TACE also affects Treg cells. Peripheral Treg cells were significantly increased in HCC patients and decreased by TACE [88,92]; their reduction was associated with improved clinical outcomes [93]. Among Treg cells, the effector Treg population, which exerts suppressive function, was also decreased by TACE, and post-TACE frequency in this population was associated with clinical outcome [92]. As Treg cells also highly express PD-1 in HCC patients [94], ICBs including PD-1 or CTLA-4 inhibitors might also be beneficial for HCC patients following TACE via further Treg reduction. Taken together, partial immune reconstitution due to TACE can occur, and targeting immune checkpoint molecules and immune-suppressive components, including Treg cells, would further augment anti-tumor immune responses and tumor surveillance even after successful TACE.

#### 4.2.2. Transarterial Radioembolization (TARE)

Transarterial radioembolization (TARE) using yttrium-90 (Y90) is an emerging option for treating locally advanced HCC that is not eligible for surgical resection. The method delivers Y90 via tumor-supplying arteries and provides tumor-restricting effects without damaging the non-malignant liver [95], although its long-term clinical outcome has yet to be determined. Similar to TACE, TARE also activates pro-inflammatory cytokines such as IL-6 and IL-8 [96], and baseline values of these cytokines are associated with liver function and survival [97].

A recent in-depth analysis using CyTOF in resected HCC samples who underwent TARE before resection showed that TARE induces the activation of local immune cells, including CD8^+^ T cells, CD56^+^ NK cells, and CD8^+^CD56^+^ NKT cells [98]. Notably, TARE also induces peripheral T-cell function and increases APCs [98]. Furthermore, PD-1^+^TIM-3^+^CD8^+^ T cells were observed in TARE responders, indicating that further enhancement of T cell responses might be feasible by using ICBs following TARE.

## 5. Clinical Studies of ICBs as an Adjuvant Treatment Following LRTs

Based on previous observations, several pilot studies have evaluated the possibility of LRTs combined with ICBs in patients with HCC. In a single-center retrospective study, LRTs, including TACE or TARE combined with nivolumab, were tolerated in a small number of patients with intermediate or advanced HCC without deterioration of liver function [99], indicating that LRTs with ICBs is a safe strategy. In another retrospective study, TARE combined with ICBs was shown to be safe and tolerable [100]. Recently, few HCC patients were also treated with RFA combined with the anti-CTLA-4 inhibitor tremelimumab, and the protocol was shown to be safe and feasible [101]. Several clinical trials are currently being conducted to evaluate the safety and efficacy of each LRT with ICB and are summarized in Table 1.

ICBs as adjuvant therapies following LRTs are currently being investigated in phase III trials. Anti-PD-L1 combined with anti-VEGF agents might also be a promising strategy as adjuvant therapy following LRTs. Durvalumab (anti-PD-L1) plus bevacizumab (anti-VEGF) following TACE (EMERALD-1) or curative treatments including RFA and surgical resection (EMERALD-2) is being evaluated as a phase III, randomized, double-blind, placebo-controlled, multicenter trial. Primary endpoints are PFS (EMELALD-1) or recurrence-free survival (EMERALD-2). The estimated primary completion year would be 2022 for EMERALD-1 and 2023 for EMERALD-2. Atezolizumab plus bevacizumab is also under the open-label, randomized, phase III trial in patients who underwent curative treatments (IMbrave050), and the estimated primary completion year would be 2023. Furthermore, safety and efficacy (PFS and OS) of pembrolizumab plus multikinase inhibitor lenvatinib following TACE is currently being investigated (LEAP-012), and the estimated primary completion year would be 2025. Furthermore, combined regimen, nivolumab plus ipilimumab is also being investigated in an adjuvant setting following TACE (CheckMate 74W), and the primary endpoint is time-to-progression. The estimated primary completion year would be 2026. Combined LRTs and ICBs could be a breakthrough for treating HCC and innovative improvement in the prognosis of HCC patients.

## 6. Concluding Remarks

Taken together, the evidence indicates that LRTs can modulate the anti-tumor immune responses in HCC patients, and the combination of LRTs and ICBs might have a synergistic effect on anti-tumor immune responses and clinical outcomes. Technically successful LRTs remain the most important because incomplete LRTs can induce angiogenesis in residual tumor cells [102]. Several clinical trials are actively underway and may offer a paradigm shift in the current HCC treatment algorithm. However, several aspects of this process remain unclear, including the timing of ICBs is uncertain, such as whether it should be administered concomitantly to the LRTs or after the LRTs. Furthermore, the time points following LRTs of the using ICBs need to be elucidated. Biomarkers that indicate those who can benefit from this combined strategy should also be developed. Consequently, detailed evaluation of immunological changes following LRTs, including in-depth immunoprofiling, is needed. To maximize the outcome when using this strategy, multidisciplinary cooperation among hepatologists, radiologists, radio-oncologists, and medical oncologists is also important.

Novel ICBs such as anti-TIM-3, anti-TIGIT, or anti-LAG-3 were also shown to restore the function of tumor-infiltrating T cells in vitro [21], and they are now under the early stage of clinical trials. Targeting TAM polarization and recruitment would also be a promising strategy. For example, the blockade of CSF1/CSF1R pathway [103] and CCL2/CCR2 axis [104] prevented TAM recruitment and improved anti-tumor immunity. CAR-T therapy, oncolytic virotherapy, cancer vaccines, and adoptive cell therapies using NK cells, NKT cells, or γδ T cells are also under investigation as novel immunotherapeutics. Whether these novel strategies would be synergistic with the LRTs also needs to be investigated.

## Figures and Tables

**Table 1:** Clinical trials of adjuvant immune checkpoint blockades following locoregional treatments for HCC.

LRTs	Adjuvant Agents	Phase	Clinical Trial Number
RFA/Cryoablation	Durvalumab + Tremelimumab	2	NCT02821754
RFA/MWA (+surgical resection)	Pembrolizumab	3	NCT03867084
RFA/MWA (+surgical resection)	Nivolumab	3	NCT03383458
RFA/MWA (+surgical resection)	Durvalumab + Bevacizumab	3	NCT03847428
RFA/MWA (+surgical resection)	Atezolizumab + Bevacizumab	3	NCT04102098
RFA/Cryoablation/TACE	Tremelimumab	1/2	NCT01853618
TACE	Durvalumab + Tremelimumab	2	NCT02821754
TACE	Durvalumab + Tremelimumab	2	NCT03638141
TACE	Pembrolizumab	2	NCT03397654
TACE	Nivolumab	2	NCT03572582
TACE	Durvalumab	3	NCT03778957
TACE	Durvalumab + Bevacizumab	3	NCT03778957
TACE	Nivolumab	3	NCT04340193
TACE	Nivolumab + Ipilimumab	3	NCT04340193
TACE	Pembrolizumab + Lenvatinib	3	NCT04246177
TACE using drug-eluting beads	Nivolumab	3	NCT04268888
TARE	Pembrolizumab	1	NCT03099564
TARE	Nivolumab	1	NCT02837029
TARE	Nivolumab	2	NCT03380130
TARE	Nivolumab	2	NCT03033446

HCC, hepatocellular carcinoma; RFA, radiofrequency ablation; MWA, microwave ablation; TACE, transarterial chemoembolization; TARE, transarterial radioembolization.

## Data Availability

Data is contained within the article.
